# Hierarchical graphical model reveals HFR1 bridging circadian rhythm and flower development in *Arabidopsis thaliana*

**DOI:** 10.1038/s41540-019-0106-3

**Published:** 2019-08-12

**Authors:** Zhana Duren, Yaling Wang, Jiguang Wang, Xing-Ming Zhao, Le Lv, Xiaobo Li, Jingdong Liu, Xin-Guang Zhu, Luonan Chen, Yong Wang

**Affiliations:** 1 CEMS, NCMIS, MDIS, Academy of Mathematics and Systems Science, Chinese Academy of Sciences, Beijing, 100190 China; 2 University of Chinese Academy of Sciences, Beijing, 100049 China; 3 State Key Laboratory of Molecular Plant Sciences and Center of Excellence for Molecular Plant Sciences, Chinese Academy of Sciences, Shanghai, 200032 China; 4 Division of Life Science, Department of Chemical and Biological Engineering, Center of Systems Biology and Human Health, State Key Laboratory of Molecular Neuroscience, The Hong Kong University of Science and Technology, Hong Kong, China; 5 Institute of Science and Technology for Brain-Inspired Intelligence, Fudan University, Shanghai, 200433 China; 6 Key Laboratory of Computational Neuroscience and Brain-Inspired Intelligence, Ministry of Education, Shanghai, China; 7 Bayer U.S. – Crop Science, Monsanto Legal Entity, St. Louis, MO 63156 USA; 8 Department of Plant Biology, Carnegie Institution for Science, 260 Panama Street, Stanford, CA 94305 USA; 9 Key Laboratory of Systems Biology, Center for Excellence in Molecular Cell Science, Institute of Biochemistry and Cell Biology, Chinese Academy of Sciences, Shanghai, 200031 China; 10 Center for Excellence in Animal Evolution and Genetics, Chinese Academy of Sciences, Kunming, 650223 China; 11 School of Life Science and Technology, ShanghaiTech University, Shanghai, 201210 China; 12 Research Center for Brain Science and Brain-Inspired Intelligence, 201210 Shanghai, China

**Keywords:** Dynamic networks, Plant sciences

## Abstract

To study systems-level properties of the cell, it is necessary to go beyond individual regulators and target genes to study the regulatory network among transcription factors (TFs). However, it is difficult to directly dissect the TFs mediated genome-wide gene regulatory network (GRN) by experiment. Here, we proposed a hierarchical graphical model to estimate TF activity from mRNA expression by building TF complexes with protein cofactors and inferring TF’s downstream regulatory network simultaneously. Then we applied our model on flower development and circadian rhythm processes in *Arabidopsis thaliana*. The computational results show that the sequence specific bHLH family TF HFR1 recruits the chromatin regulator HAC1 to flower development master regulator TF AG and further activates AG’s expression by histone acetylation. Both independent data and experimental results supported this discovery. We also found a flower tissue specific H3K27ac ChIP-seq peak at AG gene body and a HFR1 motif in the center of this H3K27ac peak. Furthermore, we verified that HFR1 physically interacts with HAC1 by yeast two-hybrid experiment. This HFR1–HAC1–AG triplet relationship may imply that flower development and circadian rhythm are bridged by epigenetic regulation and enrich the classical ABC model in flower development. In addition, our TF activity network can serve as a general method to elucidate molecular mechanisms on other complex biological regulatory processes.

## Network biology: transcription factor activity network reveals novel pathway

Transcription factor and its combinatorial regulations are fundamentally important in coordination among complex regulatory processes. An international team of researchers led by Yong Wang and Luonan Chen at Chinese Academy of Sciences proposed a novel hierarchical graphical model to infer TF activity network from its mRNA expression, protein co-factors, and downstream regulations. The TF mediated genome-wide gene regulatory network reveals HFR1-HAC1-AG pathway regulating circadian rhythm and flower development in Arabidopsis thaliana. This HFR1-HAC1-AG pathway may enrich the classical ABC model in flower development and imply epigenetic regulation via circadian rhythm. The TF activity network can serve as a general method to elucidate molecular mechanisms on other complex biological regulatory processes.

## Introduction

To study coordination among complex regulatory processes, it is necessary to go beyond individual regulators, *cis*-elements, and target genes (TGs) to provide a global connectome. Network biology holds great potential in unveiling regulatory mechanisms as previously demonstrated.^1–3^ A wide variety of approaches can take advantage of the increasingly available gene expression data to infer gene regulatory network (GRN), including ordinary and stochastic differential equations (ODEs/SDEs)-based methods,^4–8^ Bayesian and dynamic Bayesian networks (BNs/DBNs),^9–12^ and information theoretic or correlation-based methods.^13–15^ One fundamental mechanism of gene regulation is that transcription factors (TFs) serve as the key regulators for many important biological processes. TFs bind to specific DNA sequences and thereby regulate the transcription of their TGs. In addition, TFs are known to facilitate biological functions based on its protein/complex activity or interacting network rather than simply on its mRNA concentration, i.e., the mRNA expression of a TF does not represent the activity of this TF on TGs. This fact invalidates many pure gene expression based TF network reconstruction methods. The physical reality is that TF’s activity is related to its many protein cofactors during posttranscriptional and posttranslational modifications.^16^ Here we define TF activity (TFA) as DNA-binding fraction (the active part and the TF in regulating status) of each TF. This concept widely considers both the mRNA level and posttranslational regulation, such as differential splicing, posttranslational modification, and even the methylation states of the TFs.

Cases have been reported that extensive posttranslational modification on major factors or regulators frequently happens in plant and their expression as well as activity do not agree with each other well.^17^ This will affect the transcriptional networks mediated by TFs in the developmental processes of multicellular organisms by specifying when and where genes are expressed. For example, flower development is one of the most important plant developmental process. During flower development, gene expression is precisely regulated both in time and space, which then leads to the formation of flower organ. Flower development has long been known to be triggered by circadian rhythm.^18^ In *Arabidopsis thaliana*, a circadian clock, controlled by LATE ELONGATED HYPOCOTYL (LHY) and CIRCADIAN CLOCK ASSOCIATED 1, was reported to promote flowering specifically under long days by controlling the genes GIGANTEA (GI), CONSTANS (CO), and FLOWERING LOCUS T (FT).^19,20^ Later on, the GRN related to flower development has been constructed^21,22^ and many regulators were identified known to function also in circadian rhythm,^23,24^ which sheds the light on the potential connection between flower development and the circadian rhythm. However, the molecular mechanism of how circadian rhythm links flower development remains largely unexplored.

In this paper, we aim to reveal the associations between flower development and circadian rhythm at the network level, which not only identifies their new regulators or interactions but also opens a new way to study such complex processes. To dissect the molecular mechanism of TFA, we propose a model-based computational method to simultaneously infer the TFA as a hidden variable and reconstruct the TF regulatory network. Our model fully utilizes the upstream and downstream regulation information around TF and integrates the functional linkage data and gene expression data. The upstream information of a TF is its protein (cofactor or kinase)–TF interactions. The downstream information of a TF is its target gene regulations. By systematically mining the consistency of regulatory relationships with functional linkage and gene expression data, our model constructs the TF network by estimating TFA with high accuracy, which can reveal those key TFs even without differential expressions (or “dark” TF).

We reconstructed GRM connecting Flower Development and Circadian Rhythm processes (FDCRNet), and analyzed its topological structure and revealed two hubs in the network bridging these two important processes. We successfully detected the well-known factor LHY. In addition, we found an important factor, HFR1, modulating flower development. Our TFA network analysis revealed that HFR1 modulates interactions among the ABC model genes. Experimental and computational results together show that HFR1 regulates flowering, specifically the differentiation of stamens and carpels, by modulating the regulatory interaction between HAC1 and AG. Furthermore, we hypothesize that HFR1 may play an important role in the interplay between light and temperature.

## Results

### Constructing gene regulatory network with TF activities

We propose a probability framework known as a hierarchical model to construct the network. The primary goal is to predict transcriptional regulations between TFs and TGs and further use network to confer cellular states and modulate the biological activities within a cell. Since the mRNA expression levels for both TFs and TGs can be measured simultaneously by microarray or RNA-seq, pooling large amount of data and investigating TF–TG’s correlation is a straightforward way to infer possible regulatory interactions. However, the mRNA levels of the TFs cannot reflect the activity of TFs.^25,26^ For example, posttranslational modifications may alter TFs activity by affecting the secondary structure, altering affinity, increasing or decreasing protein degradation, nuclear occupancy, and DNA binding.^27^ For example, it is known that HFR1 is ubiquitinated by COP1 E3 ligase and marked for posttranslational degradation during photomorphogenesis.^28^ In addition, TF–TF heterodimer and TF-protein cofactors would affect the ability of TF binding to the DNA. Here, we computationally model TFA and interpret gene expression process in a more precise way at the genomic scale.^29^ Specifically, our hierarchical mixture model treats TFA as a hidden variable and estimates it by observing regulatory network structure and gene expression profiles, i.e., the functional concentration of each TF, termed TFA, is inferred from its gene expression data, cofactors’ expression data, and TGs’ expression data. Then correlating the TFA with expression level of TGs will inform us which TG is more likely to be regulated by a TF.

**Fig. 1:**
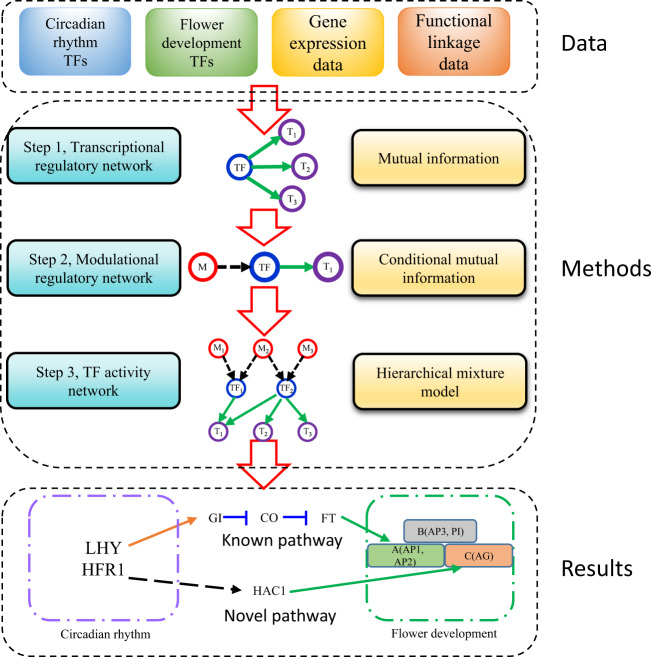
TFA network (FDCRNet) construction workflow for Flower Development and Circadian Rhythm processes. Key molecules (TFs) for circadian rhythm and flower development are curated from GO annotation database. Functional linkage and gene expression data are integrated step-by-step to construct the transcriptional regulation network, modulation regulatory network, and TFA network for those key molecules

We take an innovative approach for easier inference of an accurate network structure. We first compile a draft network by inferring pairwise and triplet regulatory relationships from correlation and functional linkage data (Step 1 and Step 2 in Fig. 1). Then this draft/candidate network is treated as prior information and updated based on the observed experimental data. TFA is introduced as a hidden variable and determined during the refinement of network structure (Step 3 in Fig. 1).

#### Step 1: Pairwise transcriptional regulations by mutual information

To identify all possible TGs for TFs and further construct TFA network, we constructed the transcriptional regulatory network based on mutual information for all possible TF–TG pairs (Materials and Methods). We fit a t-distribution using mutual information on all possible TF–TG pairs (mean μ), and only select the significant TF–TG pairs (H0: MI (TF,TG) = μ, H1: MI (TF,TG) > μ, *p*-value < 10E-4). Totally, we inferred 4234 candidate TGs for flower development (associated with 97 flowering related TFs) and 1936 candidate TGs for circadian rhythm (associated with 44 circadian-related TFs) (Supplementary Table 3 and 4). The results show that the frost tolerance gene CBF3, histone acetylation-associated gene HAC1, and the flower development-associated gene AG constitute a clique, which means each two genes are connecting. Histone acetyltransferases are known to play an important role both in flowering^30^ and cold-induced gene regulation.^31^ Interactions among these three genes reveal that histone acetylation may be bridging temperature and flowering.

#### Step 2: Modulatory triplet by conditional mutual information

**Fig. 2:**
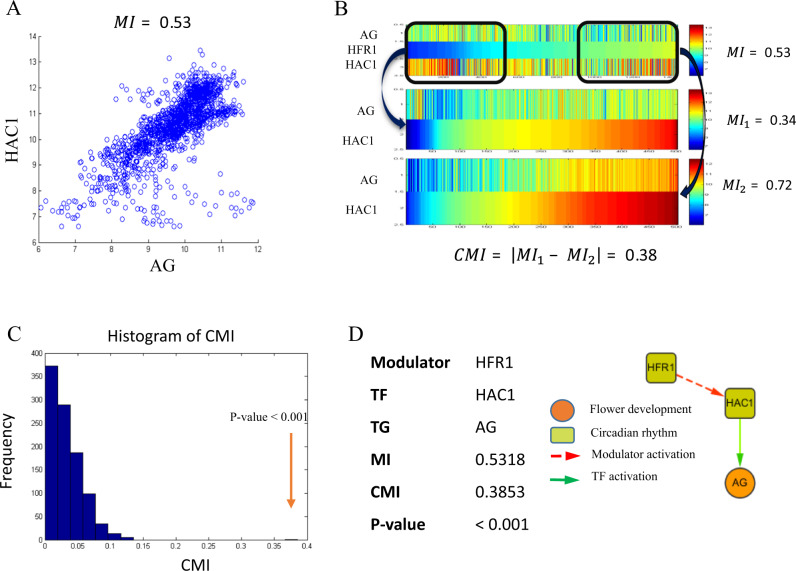
Modulators affect the pattern of the transcriptional regulation. **a** Expression pattern of HAC1 and AG. Each dot corresponds to the gene expression of HAC1 and AG in one sample. MI represents the mutual information score. **b** Top, expression pattern of a modulator–TF–target triplet: HFR1, HAC1, and AG. Each column represents a sample, which is sorted according to the expression level of the modulator (HFR1). The mutual information (MI) score of the AG and HAC1 on all the samples is 0.53. Middle, expression pattern of TF-target (HAC1 and AG) in HFR1 lowly expressed samples (bottom 35% samples). MI score is 0.34. Bottom, expression pattern of TF-target (HAC1 and AG) in HFR1 highly expressed samples (top 35% samples). TF and target gene are strongly correlated across those samples and the MI score is 0.72. The conditional mutual information (CMI) score is 0.38. **c** Significance test of the conditional mutual information of the HFR1-HAC1-AG. We randomly chose two groups of samples (each group contains 35% samples) and computed the mutual information between TF and target (HAC1-AG) in each group. We repeated this procedure 1000 times to generate the null distribution. Given HFR1’s expression level, the mutual information of HAC1 and AG is significantly changed (*p*-value < 0.001). **d** A summary for the example of the HFR1-HAC1-AG

We next considered the modulator, TF, TG triple regulatory relationships, and constructed the modulation regulatory network by conditional mutual information (CMI). The aim is to identify all the possible modulators of the TF–TG pairs for further constructing TFA network. Given a TF and its target gene pairs, we restricted their candidate modulators in the cofactors gene set which interact with TF in functional linkage network from STRING database. We computed the CMI for all possible cofactors–TF–TG triplets to identify potential modulators for each TF. Given a TF–TG pair, we generate the distribution of CMI of a random cofactor. Specifically, we randomly permute the expression data of the candidate cofactors 1,000 times, and compute CMIs on those permuted data. We test whether or not the candidate cofactor is random one by comparing the CMI with the empirical distribution of the random cofactor’s CMI (*p*-value cutoff is 0.001). Given a TF (TF-k is noted as TF_k_, and its m TGs are noted as TG_k1_,TG_k2_, …TG_km_), the *i-th* cofactor of this TF (noted as CF_ki_) is identified as a modulator if CMI(CF_ki_,TF_k_,TG_kj_) is significant for at least 50% of the TG_kj_(*j* = 1, 2, … m). As a result, we inferred 81,253 modulator–TF–TG triplets for flower development and 27,150 triplets for circadian rhythm process (Supplementary Tables 5 and 6). For example, we found that HFR1 is a modulator between HAC1 and AG. When the expression level of HFR1 is low, the mutual information of HAC1 and AG is 0.34. However, when HFR1 has a high expression level, the mutual information of HAC1 and AG is 0.72 (Fig. 2). The significant increase of mutual information correlates well with the expression change of HFR1.

#### Step 3: Integrating upstream and downstream regulations in TF activity network

**Fig. 3:**
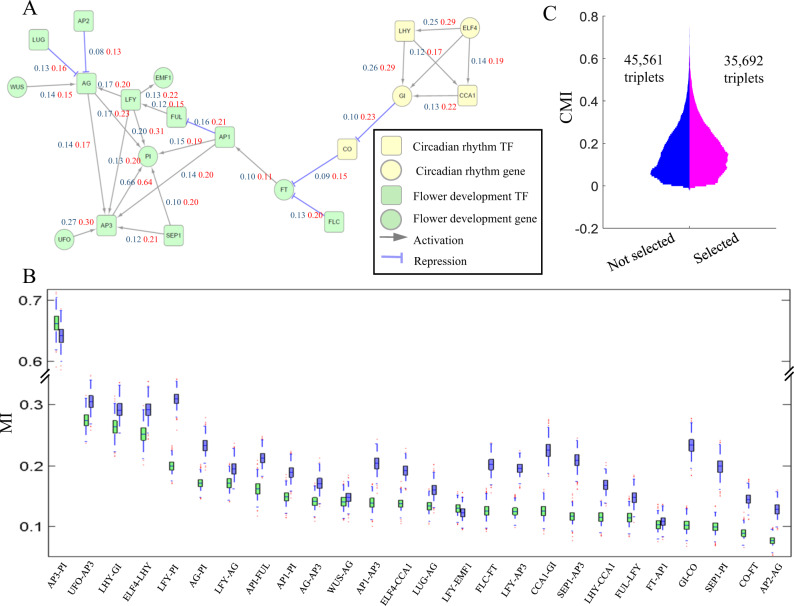
TF activity is more accurate than the TF’s mRNA expression. **a** A gene regulatory network for flower development and circadian rhythm by correlating TF and target genes. The blue score and red score on edges represent the mutual information computed by TF’s mRNA expression and TF activity, respectively. **b** Comparison of the distributions of the mutual information computed by mRNA expression and TF activity on each edge of the standard network. The green box and blue box represent the distributions of the mutual information computed by mRNA expression and TF activity, respectively. **c** Comparison of conditional mutual information on two groups of Modulator–TF–TG triplets, selected by TF activity or by TF’s mRNA expression. Note that all triplets used in comparison have significant conditional mutual information

We deduced the TFA network by integrating the upstream and downstream regulatory information. We treat the draft network structure obtained by Step 1 and Step 2 as input and estimate the TFA and the regulatory strength parameters (see Methods for details). If the estimated TFA is reliable, it should better correlate with the expression of TGs. To verify the inferred TFA, we collected 26 known TF–TG interactions in flower development process^21,32^ (Fig. 3a). On these 26 pairs, we compared two mutual information scores, one is based on mRNA expression data and the other one is based on estimated TFA. Mutual information computed by TFA is higher than that computed by mRNA expression on 92.31% (24 out of 26) of the known interactions (Fig. 3c). For example, the mutual information of GI and CO mRNA expression is 0.10, but the mutual information of GI mRNA expression and CO TFA is 0.23. To evaluate the significance of the mutual information score improvement, we generate the null distribution of the mutual information by resampling the gene expression data and TFA data 1000 times by bootstrap method (Fig. 3b). For 92.31% (24 out of 26) of the known interactions, the mutual information computed by the TFA is significantly higher than that computed by the mRNA expression. This result suggests that the TFA generates biologically plausible correlations and serves as a powerful platform for the follow-up hypothesis generation.

Overall 35,692 out of 81,253 modulator–TF–TG triplets (43.93%) were selected by TFA network (triplets with non-zero α and β). To see whether the TFA network provides additional information on CMI or not, we compared the distribution of CMI on the triplets selected by TFA model with those not selected based on TF’s mRNA expression (Fig. 3c). We find that 52.27% of the high CMI (>0.2) triplets were not selected by TFA network. The result shows that our TFA network provides additional information on MI and CMI computation, which can further filter the spurious interactions.

### Genome-wide TF activity network for flower development and circadian rhythm

We applied the TFA network reconstruction method in the 1436 samples from the TAIR database and reconstructed the genome-wide TFA network for 97 flower development-associated TFs and 44 circadian rhythm-associated TFs (Supplementary Tables 1 and 2). There are 4,556 nodes (including 664 modulators, 116 TFs, and 3,939 TGs) and 8838 edges (including 897 modulation edges and 5941 are transcriptional regulations edges) in TFA network (Supplementary Fig. 2; Supplementary Table 7).

We perform the topological analysis on the inferred TFA network. About 75% (497 out of 664) of the modulators have only one downstream TF. The distribution of the rest 25% modulator out-degree is shown in Supplementary Fig. 2b. MGP, a nuclear-localized putative TFs known to be expressed in flowering stage,^33^ modulates 64 TFs including 48 flower development TFs and 16 circadian rhythm TFs. Interestingly, both kinase WEE1 and the kinase activity-associated factor FUS5 are hub modulators. This shows that a kinase may modulate the transcriptional regulation by affecting the TF’s activity or its ability to bind the TGs. RING1B, a member of the polycomb repressive core complex 1, modulates many regulations in both FDCRNet. This chromatin remodeling enzymes may modulate the transcriptional regulations by affecting the chromatin state of TF or TGs.^34^ Some flower development or circadian rhythm-associated genes, such as HFR1 and AGL28, also have many downstream TFs. A total of 121 TFs are contained in this three-layer network, which consists of 87 flower development TFs and 42 circadian rhythm TFs. In which, 8 TFs are overlapped with flower development TFs. As a median layer, TFs are modulated by modulator and regulate TGs. The in-degrees of some important TFs are high, such as the circadian clock core TF, LHY, have 468 modulators (Supplementary Fig. 2c). The flower development TFs, AP3, and JAG, also have more than 50 modulators. HY5, associated with both FDCRNet, has a high in-degree. The circadian rhythm-associated factor COL9, ERP1, and PRR3 have a high out-degree. The key regulators of flower development, AP1, AP2, AP3, SEP2, and LFY have high out-degrees and regulate many TGs (Supplementary Fig. 2).

### Hub TFs connect flower development and circadian rhythm

**Fig. 4:**
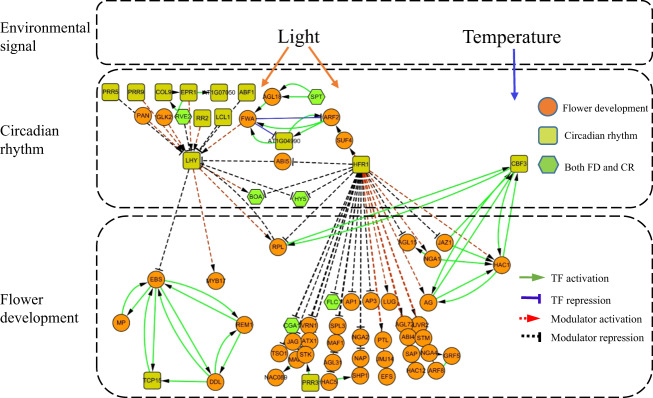
The core subnetwork among TFs in FDCRNet

Flower development and circadian rhythm are known to be closely connected,^20^ and this is demonstrated by the closeness of flower development and circadian rhythm in our global network. We then ask which regulators are central to connect the two biological processes at molecular level. To do this, we analyze a core TFA network in which all the nodes were restricted to TFs (Fig. 4). We calculated the node betweenness in this core network, which is defined as the number of the shortest paths from all nodes to all others that pass through that node. We found two nodes with the largest betweenness scores in our network: LHY and HFR1. In our network, LHY modulates 5 TFs and is modulated by 467 proteins, including 11 flower development and circadian rhythm-associated TFs. Most of the TFs modulating LHY are circadian rhythm associated, and all the TFs modulated by LHY are flower development associated. This network structure reveals that LHY is an important regulator in the process of circadian rhythm affecting flower development which is consistent with the previous study.^19,20^

The other central TF with high betweenness score is HFR1. We found that HFR1 modulates many flower development-associated TFs (Fig. 4). HFR1 is known to be involved in circadian rhythm.^35^ Interestingly, HFR1 modulates 39 flower development-associated TFs in our network. About 40% (39 out of 97) of the flower development TFs are modulated by HFR1. It seems that HFR1 bridges circadian rhythm and flower development. We gather several evidences to show the possible mechanism of HFR1’s bridging role between circadian rhythm and flower development in the following sections.

### HFR1 extends the flower development ABC model in a context specific way

**Fig. 5:**
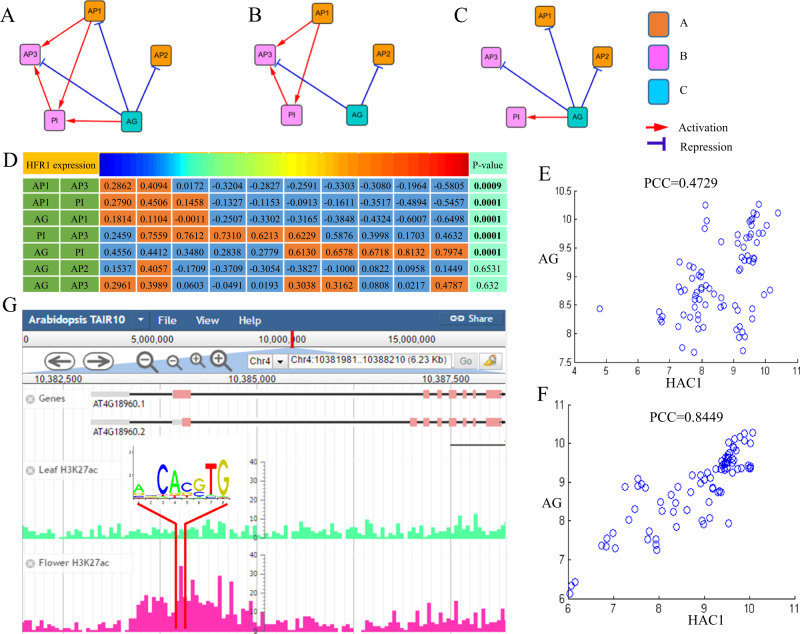
HFR1 is an important regulator for flower development and extends ABC model. **a** The known regulatory interactions within the ABC model (AP1, AP2, AP3, PI, and AG). **b**, **c** The predicted edges among the five genes by FDCRNet under the condition that HFR1 is lowly expressed and highly expressed respectively. **d** The correlations among the five genes in ABC model are dynamically changed along with the expression of HFR1. We binned the samples into ten groups by HFR1’s expression level. We then computed the Pearson correlation coefficient (PCC) among the five genes in each group. Given two genes, we binned the PCCs in ten groups into two categories, low and high, by k-means clustering. The high PCC categories are colored in orange and low PCC categories are colored in blue. The *p*-values of conditional mutual information are computed in each category by the permutation test. **e**, **f** Validation of the modulation relationship (HFR1-HAC1-AG) in an independent floral organ data. Consistent with the FDCRNet results, the correlation between HAC1 and AG is strengthened by HFR1’s high expression. **g** Comparison of H3K27ac signal of AG on flower and leaf tissues

Next, we examine the specific role of HFR1 in flower development. The flower morphogenesis has been modeled with an ABC model, with A specifying sepals, A&B petals, B&C stamens, and C carpels. Figure 5a shows the seven known interactions among the five genes in ABC model (A (AP1, AP2), B (AP3, PI), C (AG)). If HFR1 is an important regulator during flower development, it may have implications in the ABC model. To detect the role of HFR1 in the ABC model, we binned the samples into ten groups based on HFR1’s expression level and computed the Pearson correlation coefficient (PCC) on the seven known pairs in each group (Fig. 5d). Given two genes, we binned the PCCs in ten groups into two categories, low and high, by k-means clustering. Then we tested whether the categories are significantly correlated with HFR1 expression or not. We calculated the CMI among HFR1 and each pair of the seven known interactions. Then we permuted HFR1 expression data 10,000 times and calculated CMI to generate background distribution and test the significance. As a result, five out of seven pairs are significant with *p*-value < 0.0001. The regulatory interactions among these five genes are known^21^ (Fig. 5a). However, we can find that some interactions dynamically changed with the expression of HFR1 (Fig. 5b, c). In another words, the network rewires among the five genes with different HFR1 expressions (low or high). Interestingly, AP1 regulates AP3 and PI when HFR1 is lowly expressed (Fig. 5b). AG regulates AP1 and PI when HFR1 is highly expressed (Fig. 5c). The ABC model describes that A specify sepals, A&B specify petals, B&C specify stamens, and C specify carpels.^36^ Our results show that B connects to A when HFR1 is expressed at low level and connects to C when HFR1 is expressed at high level. These lines of evidence explain that high expression of HFR1 seems to be required in the differentiation of stamens and carpels by activating C to connect with B. Furthermore, these evidence together reveals that HFR1 seems to play an important modulation role during flowering in *Arabidopsis* as a regulator.

### HFR1 modulates flower development by histone acetylation of AG

Next, we explored HFR1’s function in flower development. In our TFA network HFR1 modulates many flower development-associated TFs on their TGs. However, the main target of HFR1’s regulation to flowering remains unknown. We speculate that it may be AG based on several reasons: (1) it is clear in our TFA network HFR1 regulate flowering gene AG via HAC1 (Fig. 4). AG is one of the most important flowering-associated TFs; (2) high-level expression of HFR1 activates the connection between C (AG) and B (PI) to promote the differentiation of stamens and carpels; (3) AG is the receptor of two out of three main flowering signal pathways. These three reasons encourage us to investigate the regulation of AG by HFR1 in the flower.

We compiled an independent gene expression dataset on floral organs to test if HFR1-HAC1 and AG regulatory triplet is context specific in flower. We computed the PCC on high HFR1 samples (top 35%) and low HFR1 samples (bottom 35%) (Fig. 5e, f). We find that HAC1 and AG are highly correlated when HFR1 is highly expressed in the flowing stage. This result in an independent data supports that HFR1 indeed regulates flowering via AG.

The next question is that how HFR1 regulates AG. One hypothesis is that HFR1 regulates AG through histone acetylation since HAC1 is a histone acetyltransferase to activate gene expression.^37^ The evidence is as follows: firstly, HFR1 is the modulator of HAC1, HAC5, and HAC12 in our network. These three genes are key players of histone acetylation. Secondly, HFR1 belongs to the bHLH TFs family. Some of the bHLH family members are reported to regulate HAC1 activity.^38^ These lines of evidence imply the relationship between HFR1 and HAC1.

To validate the relationship between HAC1 and AG on flower tissue, we compared the histone acetylation mark H3K27ac signal of AG on flower tissue and leaf tissue (Fig. 5g). Encouragingly, there is a H3K27ac peak in AG gene body region on flower tissue and no signal on leaf tissue,^39^ which shows that AG may be activated by histone acetyltransferase HAC1 on flower.

Furthermore, we performed motif scanning in the H3K27ac peak of AG on flower tissue. We found a bHLH family TF motif (Jaspar ID: MF0007.1) which matches the center of histone acetylation peak (chr4:10,384,160-10,384,167) (Fig. 5g). This implies a possible binding footprint of HFR1 in acetylated region and the cooperation between HFR1 and HAC1 in regulating flower gene AG.

### Experimental validation of the HFR1-HAC1 physical interaction

**Fig. 6:**
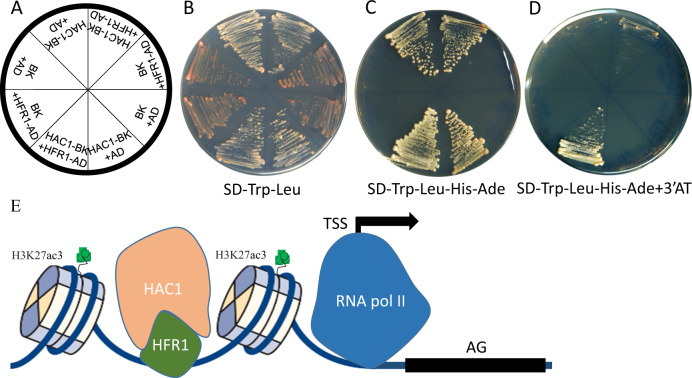
HFR1 physically interacts with HAC1 and regulates flowering by activating AG. **a** A sketch map of yeast strains on the selective plates. BD + AD, yeast strain AH109 transformed with empty vectors pGBK-T7 and pGAD-T7; HAC1-BD + AD, AH109 transformed with pGBK-T7 background vector expressing HAC1-BD and empty vector pGAD-T7; BD + HFR1-AD, AH109 transformed with pGBK-T7 empty vector and pGAD-T7 background vector expressing HFR1-AD; HAC1-BD + HFR1-AD, AH109 expressing HAC1-BD and HFR1-AD in pGBK-T7 and pGAD-T7 vectors respectively. **b**–**d** Growth of the strains streaked on selective media plates with SD/-Trp/-Leu (**b**), SD/-Trp/-Leu/-His/-Ade (**c**) and SD/-Trp/-Leu/-His/-Ade/ + 3-AT (**d**). **e** A mechanistic model for HFR1 and HAC1’s physical interaction to activate AG by histone acetylation

One simple explanation for HFR1’s modulation on HAC1’s regulation to AG is a physical interaction between HFR1 and HAC1. To test the interaction between HFR1 and HAC1, we carried out a yeast two-hybrid experiment (Fig. 6a–d). Neither the yeast strain with empty bait and prey vectors nor the strain expressing GAL4 binding domain (BD) and HFR1-AD (activation domain) fusion protein was able to grow on a synthetic dropout media (SD) plate without Trp, Leu, His, and Ade supplements, but both the strain with empty AD and HAC1-BD constructs and the strain with HFR-AD and HAC1-BD constructs could grow on the quadruple dropouts selective media, suggesting that HAC1 itself is able to activate expression of the reporter genes. To control the self-activation activity of HAC1, we added 5 mM 3-amino-1,2,4-triazole (3-AT) to the quadruple dropouts selective media. Under this condition, the strain expressing HFR-AD and HAC1-BD fusion proteins was still able to form colonies while the growth of control strain expression activation domain and HAC1-BD chimeric protein was completely inhibited. This result established that HFR1 and HAC1 are truly able to physically interact with each other.

## Discussions

We proposed a new method to infer the GRM by systematically integrating prior knowledge, i.e., TF upstream regulation and downstream regulation. There are three levels of regulatory networks reported in this study: transcription regulatory network, modulation regulatory network, and TFA network. We inferred the direct regulation (transcription regulation) and indirect regulation (modulation regulation), and at last integrated these regulations in a graphical model to get a consistent network by introducing the TFA concept.

We applied our method in two important biological processes: flower development and circadian rhythm in *Arabidopsis thaliana*. As a result, we found that two hub TFs, LHY and HFR1, bridge these two biological processes. We hypothesize that HFR1 is an important regulator of flower development in *Arabidopsis thaliana*. HFR1 modulates some important flower development regulations, such as the interactions among the ABC model genes. TFA network indicates that regulation of HAC1 on AG is modulated by HFR1. Then, we found that AG is acetylated on flower tissue and but not in leaf. Interestingly, a bHLH family TF motif (Jaspar ID: MF0007.1) occurs in H3K27ac peak near AG, which indicates the footprint of HFR1 together with histone acetylation signal. At last, we validated that HFR1 is physically interacting with HAC1 by a yeast two-hybrid experiment. These results together suggest a model that HFR1 and HAC1 physically interact and bind to AG to activate its expression via histone acetylation. More specifically, transcriptional factor HFR1 sequence-specifically binds to AG and recruits the chromatin regulator HAC1 to acetylate AG, which activates AG’s expression on flower tissue (Fig. 6e). Both experimental and public data validation support our results. These phenomena revealed that HFR1 regulates flowering, specifically regulates the differentiation of stamens and carpels by recruiting HAC1 to bind to AG, which further activates it by histone acetylation. This triplet relationship HFR1-(HAC1-AG) controlling flowering differentiation also enriches the classical ABC model in flower development.

**Fig. 7:**
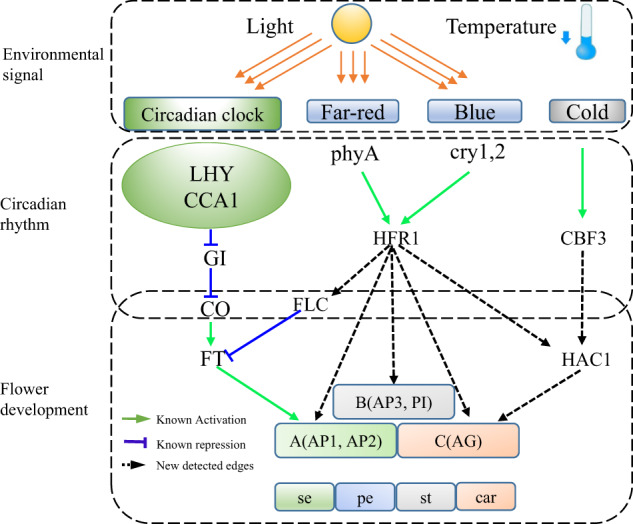
Three major pathways by which the circadian rhythm regulates flowering by environmental signature. HFR1 senses the light signal and regulates the flowering process

Our study indicates that HFR1 plays a crucial role during the interplay between light and temperature. In general, the environmental signals, such as light and temperature, promote flowering by controlling the circadian rhythm.^40,41^ Interestingly, HFR1 encodes a light-inducible protein and is involved in phytochrome signaling.^42^ We notice in our network that there are three major pathways which link environmental signals to flower differentiation (Fig. 7). The first one is a well-known pathway: light-LHY circadian clock-GI-CO-FT pathway.^32^ The second pathway is the light-HFR1 pathway to associate with some important flower development TFs. The third one is the temperature-CBF3-HAC1-AG pathway. The first pathway was reported in many studies^19,20^ and we can correctly reconstruct it by our method. The first two pathways are regulated by light^20,42,43^ and the third pathway is regulated by temperature.^20,42,43^ TF HFR1 serves a critical role in the second and third pathways. In the second pathway, HFR1 is a modulator of 39 flower development-associated TFs, including AP1, AP3, AG, FLC, and LUG. In the third pathway, HFR1 serves as a modulator between HAC1 and AG (Figs. 4–6). These evidences indicate that HFR1 plays a key role in the transducing signals from light and temperature to influence circadian and hence flowering development.

We discuss some limitations of our analysis. In our model, we did not use time ordering information, which is important in circadian rhythm, due to the following two reasons: (1) our model needs a large dataset as input data, but most of the time-course data have small number of samples; (2) we select the circadian rhythm genes from gene ontology, which have included most of the genes related to circadian rhythm. In the future, we will extend this model to consider the dynamical information^23,24^ from time-course data.

## Methods

### Sources of datasets

We sourced the key players in flower development and circadian rhythm by searching the GO database. Finally we collected 97 flower development-associated TFs and 44 circadian rhythm-associated TFs (Supplementary Table 1 and 2). We used a preprocessed gene expression data from the TAIR database in our study.^44^ The dataset contains 1436 hybridization experiments using the Affymetrix *A. thaliana* ATH1 (25 K) array, which contains 22,810 probe sets. These expression data are from multiple tissues (including leaf, root, flower, and seed) on multiple experimental conditions including biotic treatment, development, abiotic treatment, nutrient treatment, hormone treatment, chemical treatment, cell cycle, and genotype comparison. The probe IDs were converted into gene IDs. Probes that match more than one gene were removed. For those multiple probes that matched a single gene, the maximum expression value was assigned to that gene. The functional linkage data from STRING database are used to screen the candidate cofactors for the TFs.

In order to validate the relationships between HFR1, HAC1, and AG in an independent dataset, we collected public gene expression data of floral organ.^44^ This data are from 11 series (GSE5632, GSE9605, GSE9721, GSE9799, GSE15689, GSE16056, GSE2848, GSE2847, GSE27281, GSE576 and GSE607) and contain 232 samples. We used the robust multi-array average method to normalize the data. ChIP-seq data of H3K27ac on flower tissue and leaf tissue are from PlantDHS database.^39^

### Overview of methods

We introduce TFA *P* as a random variable. *D* represents the observed experimental data and *G* is the prior information from the draft network structure of the TF–TG regulations. Then the joint probability for the gene expression (*D*), TFA (*P*), and regulatory structure (*G*) can be expressed as:

$$Pr\left( {D,P,G} \right) = Pr\left( G \right)Pr\left( {D,P|G} \right)$$

Regulatory structure *G* is derived from functional linkage data and the mRNA expression correlation among TG, TF, and other cofactors. The cofactors are defined as modulator and have influences on the regulation of a TF–TG pair.^45^ Specifically, we compile a draft regulatory structure *G* by applying mutual information to detect TF–TG pairs and CMI to reveal the modulator–TF–TG triplets (Methods). Then we apply a Gaussian graphical model to estimate parameters and hidden variables by maximizing the conditional probability *Pr* (*D*, *P*|*G*).

We decomposed gene expression (*D*) into two parts, modulator expression data *Q* and target gene expression data *E*. We represent concentrations of modulators, TF activities, and expression levels of targets with random variables Q = (*Q*_1_, … *Q*_*L*_, …*Q*_*K*_), *P* = (*P*_1_, … *P*_*L*_), and *E* = (*E*_1_, … *E*_*N*_), respectively (Supplementary Fig. 1). Here, *Q*_1_, … *Q*_*L*_ are the mRNA concentrations of TFs. *Q*_*L*+1_, … *Q*_*K*_ are the concentrations of cofactors or modulators, *P* are the activities and *E* are the expressions of TGs. *Q*, *P*, and *E* are matrices of K × M, L × M, and N × M, respectively, where *M* is the number of samples. According to the dependent and conditional independent relationship in the network, we can get the likelihood as follows,

1 $$Pr\left( {Q,E} \right) = \mathop {\prod }\limits_{k = 1}^K Pr\left( {Q_k} \right)\mathop {\prod }\limits_{l = 1}^L Pr\left( {P_l|pa\left( {P_l} \right)} \right)\mathop {\prod }\limits_{n = 1}^N Pr\left( {E_n|pa\left( {E_n} \right)} \right),$$

where *pa*(*X*) represents all parents of node *X* in the network. TFA *P* is a linear combination of modulator *Q*, and the regulatory strength of the *k-* th modulator on the *l-*th TFA (TFA) is represented as β_lk_. Target gene expression *E* is fitted by linear combination of TFA *P*, and the regulatory strength of the *l-*th TF on the *n-*th target gene is represented asα_nl_. The matrix *B* = (β_lk_)_L×K_ is the effect strength of modulator on TFA and the matrix *A* = (α_nl_)_N×L_ is the regulatory strength of TF on target gene. Thus the input of TFA network inference is expression data of target gene (*E*) and modulator (*Q*). The parameters to be inferred are the regulatory strength matrix of modulator on TFA (matrix *B*), the regulatory strength matrix of TF on TGs (matrix *A*), and the hidden variables TFA (*P*).

In summary, we use a three-step procedure to reconstruct the TFA network. At first, we use the existing data to mine the possible regulations to derive the draft network structure. Step 1 and Step 2 identify the potential target and modulator for the TFs and provide the candidate connections in the network. Then in Step 3, we consider the derived network structure into Eq. (1) and estimate the parameters and hidden variables by maximum likelihood estimation (MLE) using an Expectation Maximization (EM) algorithm.

### Reconstruction of the TF activity network

Step 1, Infer transcription regulatory network. To identify the potential TGs for each TF, we detect the possible interactions by assuming that functionally related two genes are always coexpressed on the gene expression data.^46^ In this study, we use the mutual information, which measures the degree of statistical dependency between two variables to infer the transcriptional regulations. The mutual information of TF (*X*) and a potential target gene (*Y*) is defined as follows:

2 $${\mathrm{MI}}\left( {{\mathrm{X}},{\mathrm{Y}}} \right) = \mathop {\sum }\limits_{{\mathrm{x}} \in {\mathrm{X}}} \mathop {\sum }\limits_{{\mathrm{y}} \in {\mathrm{Y}}} {\mathrm{p}}({\mathrm{x}},{\mathrm{y}}){\mathrm{log}}\left( {\frac{{{\mathrm{p}}({\mathrm{x}},{\mathrm{y}})}}{{{\mathrm{p}}_1({\mathrm{x}}){\mathrm{p}}_2({\mathrm{y}})}}} \right).$$

More specifically, we apply the Gaussian–kernel estimator to estimate the mutual information. We use the optimal Gaussian bandwidth^46,47^ in Gaussian–kernel estimator.

Step 2, Infer modulation network. A modulator is a gene that influences the regulation of a TF-target gene pair.^45^ For example, TF *A* regulate target gene *B* on the condition of gene *C* is high expressed (or low expressed) but not regulate target gene B on the condition of gene *C* is low expressed (or high expressed). For this example, gene *C* is a modulator between TF A and target gene *B*. The interaction between a modulator and a TF-target regulation is defined as modulation. There are two types of interactions in a modulation network. One is TF-target regulation and the other is modulation.

With the inferred transcriptional regulations in Step 1, we then apply the CMI to detect the modulation network. MINDy, an information-theoretic algorithm, is used to estimate the CMI.^45^ The approximation of CMI of a TF (*X*) and target gene (*Y*) given a modulator (*M*) is as follows:

3 $${\mathrm{CMI}}\left( {{\mathrm{X}},{\mathrm{Y|M}}} \right) = {\mathrm{MI}}\left( {{\mathrm{X}},{\mathrm{Y|M}} \in {\mathrm{L}}_{\mathrm{m}}^ + } \right) - {\mathrm{MI}}\left( {{\mathrm{X}},{\mathrm{Y|M}} \in {\mathrm{L}}_{\mathrm{m}}^ - } \right),$$

where ${\mathrm{L}}_{\mathrm{m}}^ +$ is a set of samples on which gene M is highly expressed. Similarly, the ${\mathrm{L}}_{\mathrm{m}}^--$ represents the set of samples on which the gene M is lowly expressed.

Step 3, Infer TFA network. In Steps 1 and Step 2, we inferred the potential structure of the network. Starting from those candidates structure, we further used the observed data to estimate the TFA and then the regulatory strengths of regulations between TFs and TGs. In this step, we applied Gaussian distribution to model the conditional distribution. We assumed that random variables *Q* (modulator expression), *P* (TFA), and *E* (Target gene expression) obey Gaussian distribution with mean linear combination of the parent nodes. In this linear Gaussian model, we have the activities of TFs depending on mRNA concentrations of TFs and corresponding modulators, and thus we have

4 $$\Pr \left[ {p_{l_1}{\mathrm{|pa}}\left( {p_l} \right)} \right] \propto {\mathrm{N}}\left( {\mathop {\sum }\limits_{{\mathrm{k}} \in {\mathrm{pa}}({\mathrm{P}}_{\mathrm{l}})} {\mathrm{\beta }}_{{\mathrm{lk}}}{\mathrm{q}}_{\mathrm{k}},{\mathrm{\sigma }}_{\left( {{\mathrm{P}}_{\mathrm{l}}{\mathrm{|pa}}} \right)}^2} \right),$$

where *l* = 1, 2, ⋯ , *L*; P_l_ is the activity of *l-th* TF (hidden variables), *q*_*k*_ is the observed value of Q_k_, pa(P_l_) represent the parent nodes of P_l_, β_lk_ are the parameters to be estimated. Similarly, we have

5 $$\Pr \left[ {{\mathrm{E}}_{\mathrm{n}}{\mathrm{|pa}}\left( {{\mathrm{E}}_{\mathrm{n}}} \right)} \right] \propto {\mathrm{N}}\left( {\mathop {\sum }\limits_{{\mathrm{l}} \in {\mathrm{pa}}({\mathrm{E}}_{\mathrm{n}})} {\mathrm{\alpha }}_{{\mathrm{nl}}}{\mathrm{p}}_{\mathrm{l}},{\mathrm{\sigma }}_{\left( {{\mathrm{E}}_{\mathrm{n}}{\mathrm{|pa}}} \right)}^2} \right),$$

where *n* = 1, 2, ⋯ , *N*; *E*_*n*_ is expression of *n-th* TG, α_nl_ are the parameters to be estimated.

According to the formulae (1), (4), and (5), we have

6 $$Pr\left( {Q,E} \right) \propto \mathop {\sum }\limits_{k = 1}^K N\left( {\mu _{Q_k},\sigma _{Q_k}^2} \right)\mathop {\sum }\limits_{l = 1}^L N\left( {\mathop {\sum }\limits_k \beta _{lk}q_k,\sigma _{P_l|pa}^2} \right)\mathop {\sum }\limits_{n = 1}^N N\left( {\mathop {\sum }\limits_l \alpha _{nl}p_l,\sigma _{E_n|pa}^2} \right)$$

We need to estimate the parameter ${\mathrm{\Theta }} = \left( {{\mathrm{A}},{\mathrm{B}},{\mathrm{\sigma }}_{{\mathrm{P}}_{\mathrm{l}}|{\mathrm{pa}}}^2,{\mathrm{\sigma }}_{{\mathrm{E}}_{\mathrm{n}}|{\mathrm{pa}}}^2} \right)$ and the hidden variable *P*. To infer the optimal parameters and regulatory strengths of the graphical model, we follow the MLE to maximize the probability of observed data.^48^ We maximize the logarithm likelihood function

7 $$\begin{array}{l}{\mathrm{l}}\left( {{\mathrm{Q}},{\mathrm{E}}|{\mathrm{\Theta }}} \right) = \log \left( {{\mathrm{L}}\left( {{\mathrm{Q}},{\mathrm{E}}|{\mathrm{\Theta }}} \right)} \right) = \mathop {\sum }\limits_{{\mathrm{i}} = 1}^{\mathrm{M}} \mathop {\sum }\limits_{{\mathrm{l}} = 1}^{\mathrm{L}} {\mathrm{log}}\left( {{\mathrm{Pr}}\left( {{\mathrm{Q}}_{\mathrm{i}},{\mathrm{E}}_{\mathrm{i}}|{\mathrm{P}}_{\mathrm{l}},{\mathrm{\Theta }}} \right)} \right)\\ \quad \quad \quad \quad \quad \mathop {{\max }}\limits_{\mathrm{\Theta }} \log \left( {{\mathrm{L}}\left( {{\mathrm{Q}},{\mathrm{E}}|{\mathrm{\Theta }}} \right)} \right).\end{array}$$

We should find a “good” Θ to maximize the objective function in formula (7). Clearly, this problem belongs to the parameter estimation problem with missing data. The parameters are the regulatory strengths in the network structure and the missing data is the TFA.

### EM algorithm to estimate the parameters

We used the EM strategy to solve this problem. The EM strategy is an alternative iteration strategy. In E-step, we estimate the missing data *P* given the parameterΘ, and in M-step we estimate the parameterΘ given missing data *P*. Alternating these two steps, we get a local maximum of the logarithm likelihood function.

Given initial parameterΘ, we use hard-assignment version EM strategy. In E-step, given the parameter (candidate network structure)Θ^(i−1)^ we computed the missing data (TFA) P^(i−1)^ as follows:

8 $${\mathrm{P}}^{\left( {{\mathrm{i}} - 1} \right)} = \mathop {{\arg \max }}\limits_{\mathrm{P}} {\mathrm{log}}\left( {{\mathrm{L}}\left( {{\mathrm{P}}|{\mathrm{\Theta }}^{\left( {{\mathrm{i}} - 1} \right)},{\mathrm{Q}},{\mathrm{E}}} \right)} \right)$$

In M-step, we computed the parameter (network structure) Θ^(i)^ given the missing data (TFA) P^(i−1)^ as follows:

9 $${\mathrm{\Theta }}^{\left( {\mathrm{i}} \right)} = \mathop {{\arg \max }}\limits_{\mathrm{\Theta }} \log \left( {{\mathrm{L}}\left( {{\mathrm{\Theta }}|{\mathrm{P}}^{\left( {{\mathrm{i}} - 1} \right)},{\mathrm{Q}},{\mathrm{E}}} \right)} \right).$$

In formulae (8) and (9), the objective functions are all differentiable concave functions. Hence, the point with zero gradient is the global maximum. The solution of (8) is obtained by solving linear equations CP^(i−1)^ = D, and we have P^(i−1)^ = C^−1^D, where

$$\begin{array}{l}{\mathrm{C}} = \left( {\mathop {\sum }\limits_{{\mathrm{n}} = 1}^{\mathrm{N}} \frac{{{\mathrm{\alpha }}_{{\mathrm{ni}}}{\mathrm{\alpha }}_{{\mathrm{nj}}}}}{{{\mathrm{\sigma }}_{{\mathrm{E}}_{\mathrm{n}}|{\mathrm{pa}}}^2}}} \right)_{{\mathrm{L}} \times {\mathrm{L}}} + {\mathrm{diag}}\left( {\frac{1}{{{\mathrm{\sigma }}_{{\mathrm{P}}_{\mathrm{i}}|{\mathrm{pa}}}^2}}} \right)_{\mathrm{L}}\\ {\mathrm{D}} = \left( {\frac{{\mathop {\sum }\nolimits_{{\mathrm{k}} = 1}^{\mathrm{K}} {\mathrm{\beta }}_{{\mathrm{ik}}}{\mathrm{q}}_{\mathrm{k}}}}{{{\mathrm{\sigma }}_{{\mathrm{P}}_{\mathrm{i}}|{\mathrm{pa}}}^2}} + \mathop {\sum }\limits_{{\mathrm{n}} = 1}^{\mathrm{N}} \frac{{{\mathrm{\alpha }}_{{\mathrm{ni}}}{\mathrm{e}}_{\mathrm{n}}}}{{{\mathrm{\sigma }}_{{\mathrm{E}}_{\mathrm{n}}|{\mathrm{pa}}}^2}}} \right)_{{\mathrm{L}} \times 1}\end{array}$$

The solution of (9) is obtained by linear equations E = A^(i)^P^(i−1)^, *P*^(i−1)^ = B^(i)^Q. If P^(i−1)^ and Q have full rank, then A^(i)^ = EP^(i−1)+^, B^(i)^ = P^(i−1)^Q^+^, where X^+^ represents the pseudo-inverse matrix of the matrixx. The condition that P^(i−1)^ and Q have full rank cannot be easily satisfied. In such a case, we add a condition that the network structure should be sparse. Therefore, we have a model as follows:

10 $$\mathop {{\min }}\limits_{{\mathrm{A}},{\mathrm{B}}} \left\| {{\mathrm{E}} - {\mathrm{A}}^{({\mathrm{i}})}{\mathrm{P}}^{({\mathrm{i}} - 1)}} \right\|_2^2 + \left\| {{\mathrm{P}}^{({\mathrm{i}} - 1)} - {\mathrm{B}}^{({\mathrm{i}})}{\mathrm{Q}}} \right\|_2^2 + \left\| {{\mathrm{\lambda }}\left( {{\mathrm{A}}_1 + {\mathrm{B}}_1} \right)} \right\|$$

We use ADMM^49^ to solve this L1 regularization optimization problem. The estimation of the variance ${\mathrm{\sigma }}_{{\mathrm{P}}_{\mathrm{l}}|{\mathrm{pa}}}^2,{\mathrm{\sigma }}_{{\mathrm{E}}_{\mathrm{n}}|{\mathrm{pa}}}^2$ is as follows:

11 $${\mathrm{\sigma }}_{{\mathrm{P}}_{\mathrm{l}}\,{\mathrm{pa}}}^{2\,({\mathrm{i}})} = \frac{{\mathop {\sum }\nolimits_{{\mathrm{m}} = 1}^{\mathrm{M}} \left( {{\mathrm{p}}_{{\mathrm{lm}}}^{({\mathrm{i}} - 1)} - \mathop {\sum }\nolimits_{{\mathrm{k}} = 1}^{\mathrm{K}} {\mathrm{\beta }}_{{\mathrm{lk}}}^{\left( {\mathrm{i}} \right)}{\mathrm{q}}_{{\mathrm{km}}}} \right)^2}}{{\mathrm{M}}}$$

12 $${\mathrm{\sigma }}_{{\mathrm{E}}_{\mathrm{n}}|{\mathrm{pa}}}^{2({\mathrm{i}})} = \frac{{\mathop {\sum }\nolimits_{{\mathrm{m}} = 1}^{\mathrm{M}} \left( {{\mathrm{e}}_{{\mathrm{nm}}}^{({\mathrm{i}} - 1)} - \mathop {\sum }\nolimits_{{\mathrm{l}} = 1}^{\mathrm{L}} {\mathrm{\alpha }}_{{\mathrm{nl}}}^{\left( {\mathrm{i}} \right)}{\mathrm{p}}_{{\mathrm{lm}}}} \right)^2}}{{\mathrm{M}}}$$

### Yeast two-hybrid experiment to validate HFR1-HAC1 interaction

The yeast two-hybrid experiment was performed by using Matchmaker^TM^ GAL4 two-hybrid system 3 (Clontech Laboratories, Inc. USA). To make the expression vectors, the coding sequence of *HAC1* was de novo-synthesized and in frame inserted into the bait vector pGBK-T7 to form the express vector pGBK-HAC1. Meanwhile, the coding fragment of *HFR1* was amplified with primers 5′-CATATGATGGGGAGAGCTCCGTGC-3′ (introduced with *a NdeI* restriction site) and 5′-GTCGACAATTCATCCCAAGCTTTCCCGG-3′ (introduced with *a SalI* restriction site) from Col-0 cDNA library. The fragment was then in frame recombined into the *NdeI* and *XhoI* site of bait vector pGBK-T7 by enzyme restriction to generate the expression vector pGBK-HFR1. The yeast strain AH109 was then transformed with these vectors according to the manual of Matchmaker ^TM^ GAL4 Two-Hybrid System 3. Positive transformants were screened on selective SD/-Trp/-Leu media. Medium-stringency examination of interaction was performed on SD/-Trp/-Leu/-His/-Ade media. To minimize the leaky expression of *HIS3* that can be caused by self-activation of HAC1, 3-AT was added into the SD/-Trp/-Leu/-His/-Ade media at a final concentration of 5 mM.

### Method statement

Our method was performed in compliance with relevant guidelines and regulations and was approved by Academy of Mathematics and Systems Science, Chinese Academy of Sciences.

### Reporting summary

Further information on research design is available in the Nature Research Reporting Summary linked to this article.

## Supplementary information

SUPPLEMENTAL MATERIAL

Reporting Summary

## Data Availability

Data that support the findings of this study is from the TAIR database (https://www.arabidopsis.org/).

## Supplementary information

**Supplementary information** accompanies the paper on the *npj Systems Biology and Applications* website (10.1038/s41540-019-0106-3).

## Code availability

Code for TF activity network is in github (https://github.com/durenzn/TF-activity-network).
