# Supplementary material for: Hierarchical graphical model reveals HFR1 bridging circadian rhythm and flower development in Arabidopsis thaliana
Source: NPJ Syst Biol Appl. 2019 Aug 12;5:28. doi: 10.1038/s41540-019-0106-3 (PMC6690920; doi:10.1038/s41540-019-0106-3)
Supplement: Supplementary file 1 — SUPPLEMENTAL MATERIAL [file 41540_2019_106_MOESM1_ESM.pdf]

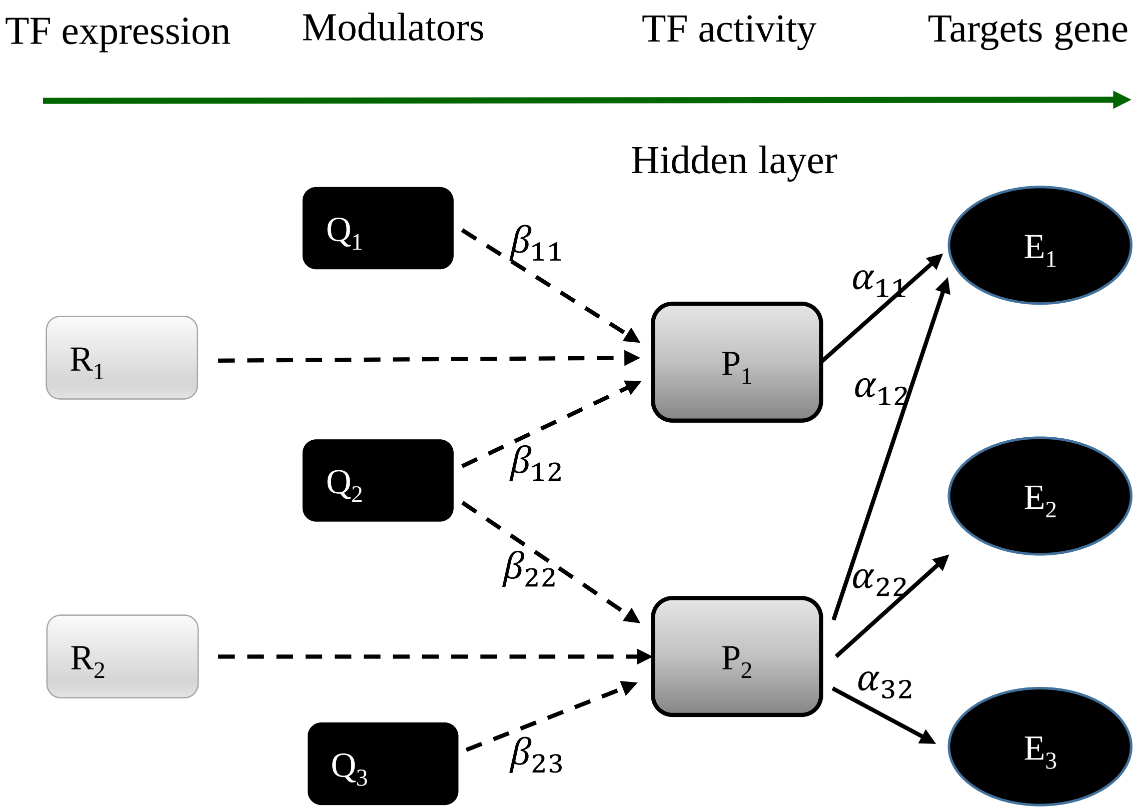

$$\Pr(R,Q,E) \propto \prod \Pr(\mathrm{R}) \Pr(Q)\, P(P|Q)P(P|R)P(E|P)$$

$$= \prod_{l=1}^{2} N(q_l, \sigma_{P_l}^2) \prod_{k=1}^{3} N(\mu_{Q_k}, \sigma_{Q_k}^2) \prod_{l=1}^{2} N(\sum_{k} \beta_{lk} q_k\, , \sigma_{P_l|pa}^2) \prod_{n=1}^{3} N(\sum_{l} \alpha_{nl} p_l\, , \sigma_{E_n|pa}^2)$$

Supplementary Figure 1

Supplementary Figure 1. A graphical model of the TF activity network. There are three observed variables: TF's expression (R), Modulator's expression (Q), and target gene's expression (E) and one hidden variable: TF's activity (P). Target gene's expression is affected by TF's activity. The activity of a TF is jointly determined by the modulators and TFs' expression.

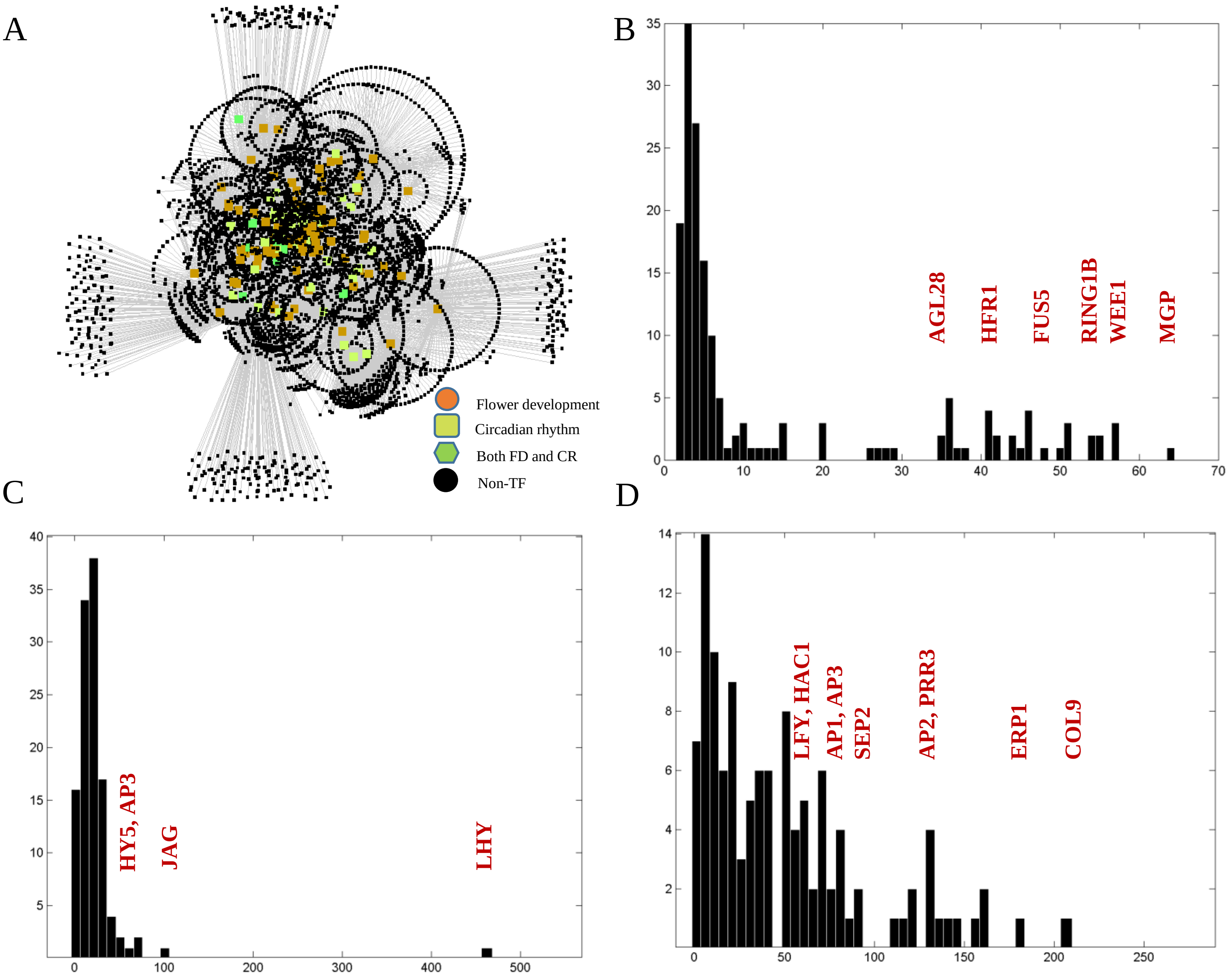

Supplementary Figure 2

Supplementary Figure 2. Topological structure of the TF activity network. (A) The FDCRNet. (B-D) The distributions of modulators' out-degree, TFs' in-degree, and TFs' out-degree.

Supplementary Tables

Supplementary Table 1. The list of the flower development associated TFs used in our FDCRNet.

Supplementary Table 2. The list of the circadian rhythm associated TFs used in our FDCRNet.

Supplementary Table 3. The transcriptional regulation network for flower development.

Supplementary Table 4. The transcriptional regulation network for circadian rhythm.

Supplementary Table 5. The modulation regulation network for flower development.

Supplementary Table 6. The modulation regulation network for circadian rhythm.

Supplementary Table 7. The integrative FDCRNet.
